# Interleukin-3 Does Not Affect the Differentiation of Mast Cells Derived from Human Bone Marrow Progenitors

**DOI:** 10.1080/08820130701741742

**Published:** 2008-01-23

**Authors:** Yuji Shimizu, Kenji Matsumoto, Yoshimichi Okayama, Sakai Kentaro, Toshitaka Maeno, Tatsuo Suga, Toru Miura, Shinji Takai, Masahiko Kurabayashi, Hirohisa Saito

**Affiliations:** ^1^ Department of Respiratory Medicine, National Hospital Organization, Takasaki Hospital, Takasaki, Japan; ^2^ Department of Medicine and Biological Science, Gunma University Graduate School of Medicine, Maebashi, Japan; ^3^ RIKEN Research Center for Allergy and Immunology, Yokohama, Japan; ^4^ Department of Nutrition and Health Promotion, Faculty of Human Life Science, Hiroshima Jogakuin University, Hiroshima, Japan; ^5^ Kirin Brewery Incorp., Takasaki, Japan; ^6^ Department of Pharmacology, Osaka Medical College, Osaka, Japan

**Keywords:** Interleukin-3, Human mast cells, Bone marrow, Stem cell factor, Differentiation

## Abstract

Although IL-3 is commonly used for culture of human progenitor-derived mast cells together with Stem cell factor (SCF) and IL-6, the effect of IL-3 on human mast cell differentiation has not been well elucidated. Human bone marrow CD34+ progenitors were cultured for up to 12 weeks in the presence of rhSCF and rhIL-6 either with rhIL-3 (IL-3 (+)) or without rhIL-3 (IL-3 (−)) for the initial 1-week of culture. Total cell number increased at 2 weeks in IL-3 (+), as compared to IL-3 (−), but changes in the appearance of mast cells were delayed. When IL-3 was present for the initial 1-week culture, granules looked more mature with IL-3 than without IL-3. However, tryptase and chymase contents, and surface antigen expression (CD18, CD51, CD54, and CD117) were not altered by IL-3. Surface expression and mRNA level of FcεRIα and histamine release by crosslinking of FcεRIα did not differ from one preparation to the next. GeneChip analysis revealed that no significant differences were observed between IL-3 (+) and IL-3 (−) cells either when inactivated or activated by aggregation of FcεRIα. These findings indicate that initial incubation of human bone marrow CD34+ progenitors with IL-3 does not affect the differentiation of mast cells.

## INTRODUCTION

IL-3 plays an important role in the growth, survival and differentiation of eosinophils (Gregory, 2003) and basophils (Zheng, 2002). In rodent models, stimulation of progenitors with IL-3 acting in synergy with SCF has been required for the development of mast cells. Through experiments with IL-3deficient mice, however, IL-3 has been shown not to be essential for the generation of mast cells, but to contribute to increased numbers of tissue mast cells, enhanced basophil production, and protective immunity against parasites, as evidenced in mice with and without a deletion of the IL-3 gene that were infected with *Strongyloides venezuelensis* (Lantz, 1998).

In humans, exogenous SCF is essential for the differentiation and activation of mast cells. IL-6 was shown to be effective in stimulating the proliferation of differentiated mast cells derived from human cord blood progenitors (Saito, 1996). However, it is controversial whether IL-3 affects the differentiation of mast cells in humans. Kirshenbaum et al. have found that IL-3 alone and in combination with SCF promoted the growth and survival of human mast cells from bone marrow progenitors (Kirshenbaum, 1992, Kirshenbaum, Kessler, Goff and Metcalfe, 1991). Yanagida et al. (1995) reported that functions in cultured cord blood mast cells could be regulated by IL-3 and its receptor. Conditioned medium from a cell line derived from a patient with mastocytosis was shown to induce the generation of mast cells that possess high affinity IgE receptors from unsorted bone marrow cells (Li, 1995), although most cells developed into mast cells that lack tryptase (Li, Meng and Krilis, 1996).

We have established a human mast cell culture system from CD34+ bone marrow progenitors. When CD34+ progenitors are cultured in the presence of rhSCF + rhIL-6, predominant cells develop into tryptase- and chymase-positive human bone marrow mast cells (HBMMC) (Shimizu, 2002). Functional FcεRIα was constitutively expressed, and its expression preceded that of tryptase during differentiation (Shimizu, 2004).

It was shown that a combination of 3 signals through gp130, c-kit and IL-3R exerted a dramatic synergistic effect on hematopoietic colony formation (Kimura, 1997). It is recommended that IL-3 at lower concentration be added only at the beginning of the culture in the human cord blood system to obtain highly purified mast cells (Saito, 2006). However, morphological, functional and molecular differences have not been well elucidated when progenitors were cultured with IL-3. We therefore investigated whether IL-3 affects the differentiation of HBMMC.

## MATERIALS AND METHODS

### Chemicals

Peroxidase-conjugated streptavidin (Biosource International, Camarillo, CA), N, N-dimethylformamide (Aldrich Chemical Company Inc., Milwaukee, WI), naphthol AS-MX phosphate, L-glutamine, levamisole, collagenase, and the AEC chromogen kit (Sigma, St Louis, MO); human serum AB (Cosmo Bio CO., LTD, Tokyo, Japan); and Ficoll-Paque research grade (Pharmacia Biotech AB, Uppsala, Sweden), were purchased commercially. Media III, which contains insulin (10 μg/ml), transferrin (10 μg/ml), HEPES (4,470 μg/ml), NaHCO_3_ (2.3 mg/ml), penicillin (100 U/ml), and streptomycin (100 μg/ml), was purchased from Immuno-Biological Laboratories, Fujioka, Japan. RhSCF was a gift from AMGEN Inc. (Thousand Oaks, CA). RhIL-3 and rhIL-6 were from Kirin Brewery Inc (Takasaki, Japan).

### Antibodies

Anti-tryptase mAb conjugated to alkaline phosphatase (G3/AP) was kindly provided by Professor Lawrence B. Schwartz (Virginia Commonwealth University, Richmond, VA). CRA-1, used for the detection of FcεRIα by either immunocytochemistry or flow cytometric analysis (Ra, 1993; Yanagihara, 1994), was purchased from Kyokuto Pharmaceutical Indust. Co. Ltd (Ibaraki, Japan). For flow cytometry, cells were incubated with several monoclonal antibodies (mAbs) recognizing various CD antigens. Most of them were obtained from the 6th International Workshop and Conference on Human Leukocyte Differentiation Antigens (Kishimoto, 1997).

Anti-human CD117 mAb (YB5.B8)(Pharmingen, San Diego, CA), anti-CD123 mAb (IL-3R, 6H6)(Serotec, Kidlington, Oxford, UK), MOPC 31C (irrelevant mouse IgG1 control), and MOPC 141 (irrelevant mouse IgG2b control) (Sigma Chemical CO., St Louis, MO), were purchased commercially. Purified human IgE and goat anti-human IgE polyclonal antibody were obtained from Chemicon (Temecula, CA).

### Cell Culture

Human bone marrow was collected in a heparinized tube from patients without any hematological disorders with informed consent. The protocol was approved at the Institutional Review Board of Gunma University. Bone marrow CD34+ cells were magnetically sorted with a CD34 progenitor isolation kit (Miltenyi Biotec, Bergisch Gladbach, Germany), as described previously (Shimizu, 2002). Sorted cells were washed, resuspended in Media III supplemented with 10% human serum AB, 2mM L-glutamine and 50 μmol/L 2-mercaptoethanol, and seeded into plastic 48-well plates (Iwaki Glass, Tokyo, Japan) at a concentration of 5 × 10^4^ cells/ml with a final concentration of 100 ng/ml rhSCF and 50 ng/ml rhIL-6.

Half of the CD34+ cells were cultured in the presence of rhIL-3 (final concentration, 10 ng/ml). Cells were cultured in 5% CO_2_ at 37°C in a humidified atmosphere. Half of the medium was exchanged once a week with the same concentration of rhSCF and rhIL-6 (no IL-3 added). A small proportion of suspended cells were stained with modified toluidine blue as described by Kimura et al. (Kimura, Moritani and Tanizaki, 1973), and cells stained metachromatically were counted by haemacytometer at each time point.

### Electron Microscopy

HBMMC at 12 weeks of culture in the presence of rhSCF+rhIL-6 either with or without IL-3 for the first week of culture were spun down at 250 × g for 8 minutes. Cell pellets were then fixed in 2.5% glutaraldehyde in 0.1% cacodylate buffer, pH 7.4, for 1 hr at 4°C. The specimens were then washed with the same buffer, and postfixed with 1% osmium tetroxide for 1 hr. The samples were dehydrated in a graded ethanol series and embedded in Epon. Thin sections were cut with an ultramicrotome, and stained with a saturated aqueous solution of uranyl acetate and lead citrate, and observed with a JEM-1200EXII (JEOL. Ltd., Tokyo, Japan).

### Measurement of Mediators and Mediator Release Assay

Different forms of tryptase (both total and β-tryptase) in HBMMC were measured using the sandwich ELISA, as described previously (Schwartz, 1994). Chymase was also measured by sandwich ELISA, as described (Takai, et al., 2000), using anti-chymase mAb (developed by the Department of Pharmacology, Osaka Medical College, Osaka, Japan) as a capture antibody, and biotinylated anti-chymase mAb (Chemicon International, London, UK) as a detection antibody.

For the quantification of histamine, HBMMC (93–99% mast cells) were sensitized overnight with or without 2 μg/ml of purified human IgE in the presence or absence of 100 ng/ml rhSCF at 37°C. Cells were transferred into centrifuge tubes, washed with Media III, and then challenged with or without 2 μg/ml goat anti-human IgE in the presence or absence of 100 ng/ml rhSCF for 30 min at 37°C. After challenge, cells were immediately spun down at 4°C, and supernatants were collected. After the removal of supernatants, cell pellets were resuspended in serum free-Media III, and lysed. The amount of histamine in supernatants and cell pellets was then quantified by radioimmunoassay with Histamine Kit (Immunotech S.A., Cedex, France), as recommended by the manufacturer. Percent histamine release was calculated as follows:

$$\%\text{histamine release}=\frac{\text{histamine in supernatants}}{\text{histamine in cell pellets}+\text{supernatants}}$$

### Real-Time Quantitative RT-PCR

Total RNA was isolated from either CD34-sorted cells, or mast cell/mast cell precursors at 2-, 4-, and 12-weeks of culture with an RNeasy mini kit according to the manufacturer’s instructions (Qiagen Inc., Valencia, CA). Forward and reverse oligonucleotide primers were constructed with Primer Express software (Applied Biosystems, Foster City, CA), and purified by Invitrogen Life Technologies (Tokyo, Japan). The primers were as follows: tryptase 5′-CAGTGGGTGCTGACCGC-3′ (nucleotide position 251), and reverse 5′-GCAGTTGCACCCTGAGGG-3′ (position 323); FcεRIα forward 5′-GCCTTACTGTTCTTCGCTCCAG-3′ (position 146), and reverse 5′-CCATGGAGGGTTCAAGGAGAC-3′ (position 220); GAPDH forward 5′-CTTCACCACCATGGAGAAGGC-3′, and reverse 5′-GGCATGGACTGTG GTCATGAG-3′. TaqMan probes for use in real time RT-PCR were also designed with Primer Express software, and prepared by Applied Biosystems.

The probe sequences for tryptase, FcεRIα, and GAPDH, respectively, were: 5′-CGGACGTCAAGGAT-3′ (position 285), 5′-TGGCGTGTTAGCAGTC CCTCAGAAACC-3′ (position 169), and 5′-CCTGGCCAAGGTCATCCATGA CAACTTT-3′. Real time RT-PCR was performed with an ABI PRISM( 7700 Sequence Detection System (Applied Biosystems Japan Ltd., Tokyo, Japan), as described previously (Shimizu, 2004).

### GeneChip Expression Analysis

HBMMC (0.5–2 × 10^6^ cells) cultured in the presence or absence of IL-3 were activated by IgE crosslinking, as shown below, and total RNA (0.5–2 μg) was extracted from inactivated or activated cells. Human genome-wide gene expression was examined using the Human Genome U133A probe array (GeneChip, Affymetrix, Santa Clara, CA), according to the manufacturer’s protocols (Affymetrix, Inc., Santa Clara, CA) as described previously (Nakajima, 2001; Okumura, 2003). Data were considered significant when the level of expression changed by at least 3-fold.

### Flow Cytometry and Cell Sorting

HBMMC at 12 weeks of culture in the presence or absence of IL-3 for the initial week of culture were washed with PBS/1% BSA (94 to 99% of cells exhibited metachromasia and were labeled with G3/AP). Cells were then incubated with several mAbs or irrevalent isotype-matched control IgG1 or IgG2b for 30min at 4°C. Cells were washed three times with PBS/BSA, and incubated with a FITC-conjugated F(ab')2 fragment of rabbit anti-mouse immunoglobulins for 30 min at 4°C.

After 3 washes with PBS/BSA, cells were resuspended in sheath fluid, and subjected to flow cytometry. Propidium iodide was added (at a final concentration of 10 μg/ml) prior to the analysis to exclude dead cells, and fluorescence was analyzed with an EPICS ELITE Flow Cytometer (Coulter, Hialeah, FL). The analysis was performed with at least 5,000 cells using WinMDI software (Coulter).

### Statistics

Statistical analyses were made with Student's *t*-test. Results were expressed as means plus or minus standard errors of the means unless otherwise indicated.

## RESULTS

1. Differentiation of human bone marrow CD34+ cells in the presence of SCF+IL-6 either with or without the initial stimulation with IL-3.

**Figure 1:**
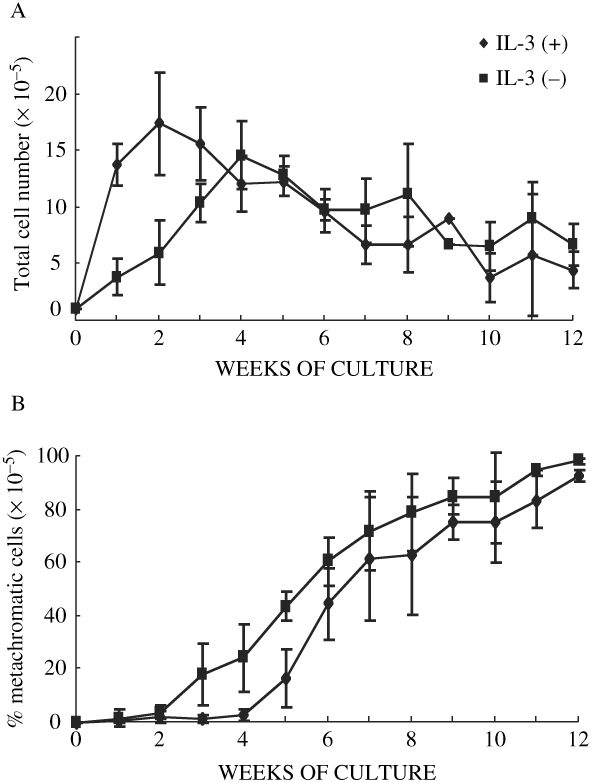
Effect of IL-3 on the differentiation of human bone marrow CD34 progenitors into mast cells. CD34+ human bone marrow progenitors were cultured with (♦) or without (▪) IL-3 for the initial 1 week in the presence of rhSCF+rhIL-6. Total cell number (A) and the percentage of metachromatic cells (B) was determined with toluidine blue stain for up to 12 weeks. Data are expressed as the mean ± SEM of five independent experiments.

CD34-sorted cells obtained from human bone marrow were cultured with or without an initial 1 week stimulation with IL-3 in the presence of rhSCF+rhIL-6 for up to 12 weeks, and total cell number and the percentage of metachromatic cells were determined by staining with toluidine blue. Cells cultured with IL-3 for the first week were designated as ‘IL-3 (+) cells’. Cells cultured without IL-3 were termed “IL-3 (−) cells.” The constant incubation with IL-3 during culture induced the differentiation of CD34+ cells into non-mast cells such as eosinophils and basophils (data not shown). As shown in Figure 1A, 1 × 10^5^ CD34+ progenitor cells increased 17.4 ± 4.5 times at 2 weeks of culture in IL-3 (+) cells, and 6.1 ± 2.8 times in IL-3 (−) cells. After 4 weeks of culture, the number of cells was not significantly different between these groups.

In spite of the temporal increase in the number of IL-3 (+) cells, 3 ± 0.8% showed metachromasia at 4 weeks of culture, whereas 24 ± 14% of IL-3 (−) cells showed metachromasia, as shown in Figure 1B. The % metachromatic cells increased along with mast cell differentiation, and maximal ratio of toluidine blue-positive cells reached more than 95% in both preparations. Cytospin preparations were also stained immunocytochemically with G3/AP, which showed similar data as toluidine blue stain (data not shown).

2. Ultrastructural appearance of HBMMC (IL-3 (+) or (−)) examined by electron microscope.

**Figure 2:**
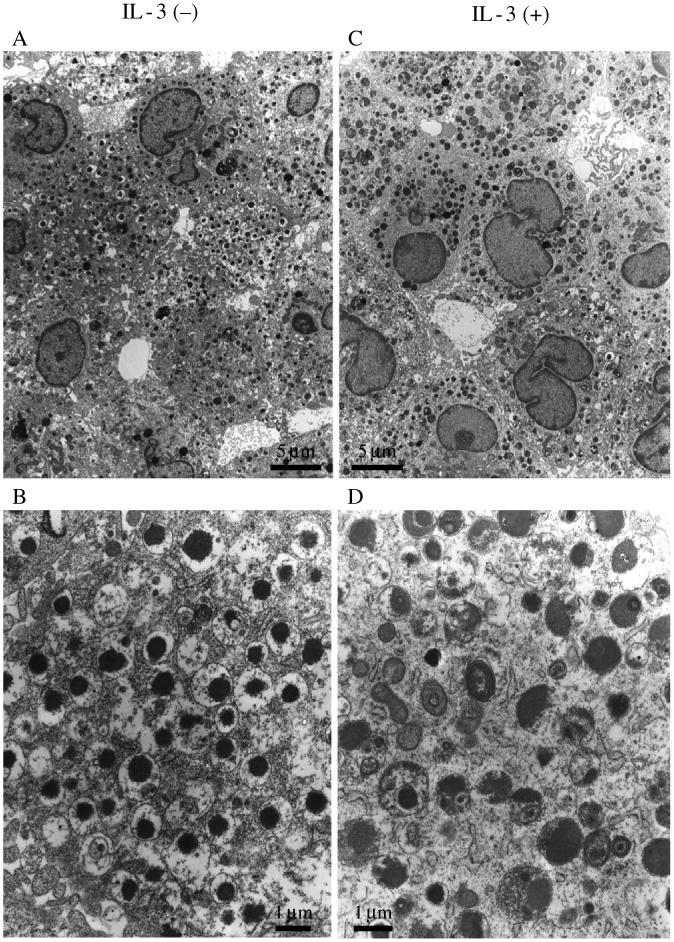
Electron microscopic appearance of 12-week-old HBMMC in the presence or absence of IL-3 for the initial 1 week of culture. Ultrastructural analysis of HBMMC cultured in the presence of rhSCF + rhIL-6 either with (A, B) or without (C, D) IL-3. Note that granules were filled with materials when IL-3 was added for the initial 1 week of culture. Representative electron micrographs are shown. Original magnification of Fig 2A and 2C is ×2,000, and Fig 2B and 2D is ×10,000.

IL-3 (+) and IL-3 (−) cells were subjected to electron microscopy. As shown in Figures 2A and 2B, each IL-3 (−) cell had a large and irregular nucleus with condensed chromatin. Almost every granule contains an electron dense core, with most of the granule remaining electron lucent, as reported previously (Shimizu, 2002). IL-3 (+) cells had a similar shaped nucleus, but the granules were filled with electron lucent materials, as shown in Figures 2C and 2D. The density of the granules in IL-3 (+) cells was lower than that in IL-3 (−) cells, and compact solid-core scrolls were partially detected, which have been shown to be present in both MCT and MCTC (Craig et al., 1988). We therefore quantified tryptase and chymase in each cell preparation.

3. Quantification of granule enzymes stored in IL-3 (+) and IL-3 (−) cells at 12 weeks of culture.

**Figure 3:**
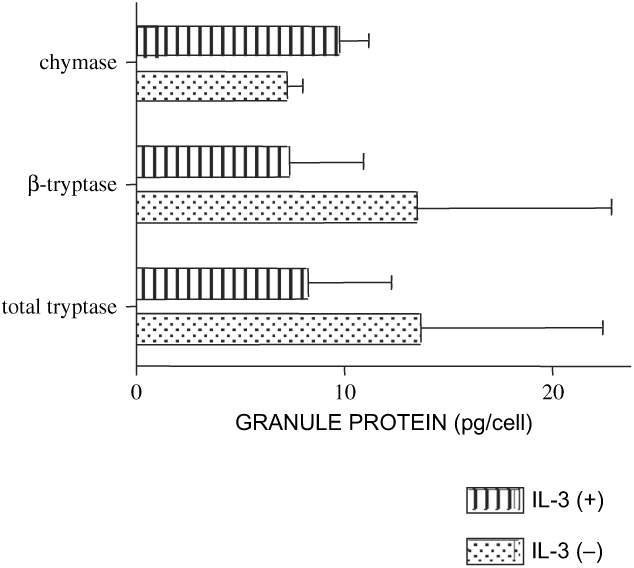
Quantification of tryptase (both total and β) and chymase in IL-3 (+) and IL-3 (−) cells cultured for 12 weeks in the presence of rhSCF + rhIL-6. IL-3 (+) and IL-3 (−) cells were collected, and pellets were sonicated. Tryptase (both total and β) and chymase were measured by ELISA. Data are the mean ± SEM of three independent experiments.

Both tryptase and chymase in mast cell granules were quantified by ELISA. As shown in Figure 3, chymase in 12-week-old IL-3 (+) cells (9.71 ± 1.45 pg/cell) and IL-3 (−) cells (7.25 ± 0.77 pg/cell) was not significantly different between IL-3 (+) and IL-3 (−) cells. Tryptase (both total and β) in IL-3 (+) (8.27 ± 3.94 and 7.34 ± 3.60 pg/cell, respectively) and IL-3 (−) (13.65 ± 8.75 and 13.49 ± 9.36 pg/cell, respectively) were also not significantly different in the two preparations.

4. Quantitative mRNA levels during differentiation.

**Figure 4:**
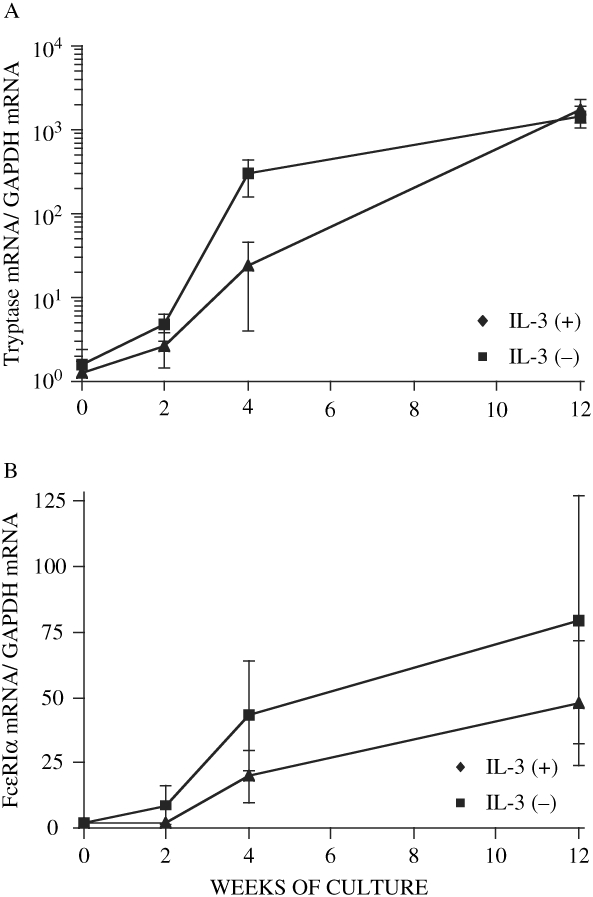
Quantitative mRNA level of tryptase and FcεRIα during differentiation. Total RNA was extracted from CD34 progenitors, and mast cell/mast cell progenitors either with or without IL-3 at 2, 4, and 12 weeks of culture. Real time RT-PCR was then performed to show the transition of the transcript of tryptase and FcεRIα. Data are the mean ± SEM of 3 independent experiments.

Percent tryptase RNA divided by GAPDH RNA in IL-3 (+) and IL-3 (−) cells (% value of mean mRNA levels as compared to 12-week-old HBMMC is also shown) was 1.3 ± 0.5 (0.07%) and 1.6 ± 0.8 (0.11%) at 0 week, 2.1 ± 0.8 (0.12%) and 5.3 ± 2.0 (0.36%) at 2 weeks, 24.7 ± 20.7 (13.9%) and 302.7 ± 141.6 (20.6%) at 4 weeks, and 1773.1 ± 574.7 and 1470.1 ± 421.8 at 12 weeks, respectively, as shown in Figure 4A. Percent FcεRIα RNA divided by GAPDH RNA in IL-3 (+) and IL-3 (−) cells was 1.9 ± 1.5 (2.4%) and 1.9 ± 1.5 (4.0%) at 0 week, 8.9 ± 7.5 (11.2%) and 2.0 ± 1.2 (4.2%) at 2 weeks, 43.1 ± 21 (54.4%) and 20.0 ± 9.8 (41.8%) at 4 weeks, and 79.3 ± 47.3 and 47.9 ± 23.9 at 12 weeks, respectively, as shown in Figure 4B. The transition of chymase transcript was similar to that of tryptase transcript (data not shown). Tryptase transcript level was reduced when progenitors were incubated initially with IL-3, but FcεRIα transcript was not significantly different between IL-3 (+) and IL-3 (−) cells. At 12 weeks, the transcript of tryptase and FcεRIα reached a similar level.

5. Surface expression of FcεRIα, CD13, CD123 and integrins on HBMMC cultured initially either with or without IL-3.

**Figure 5:**
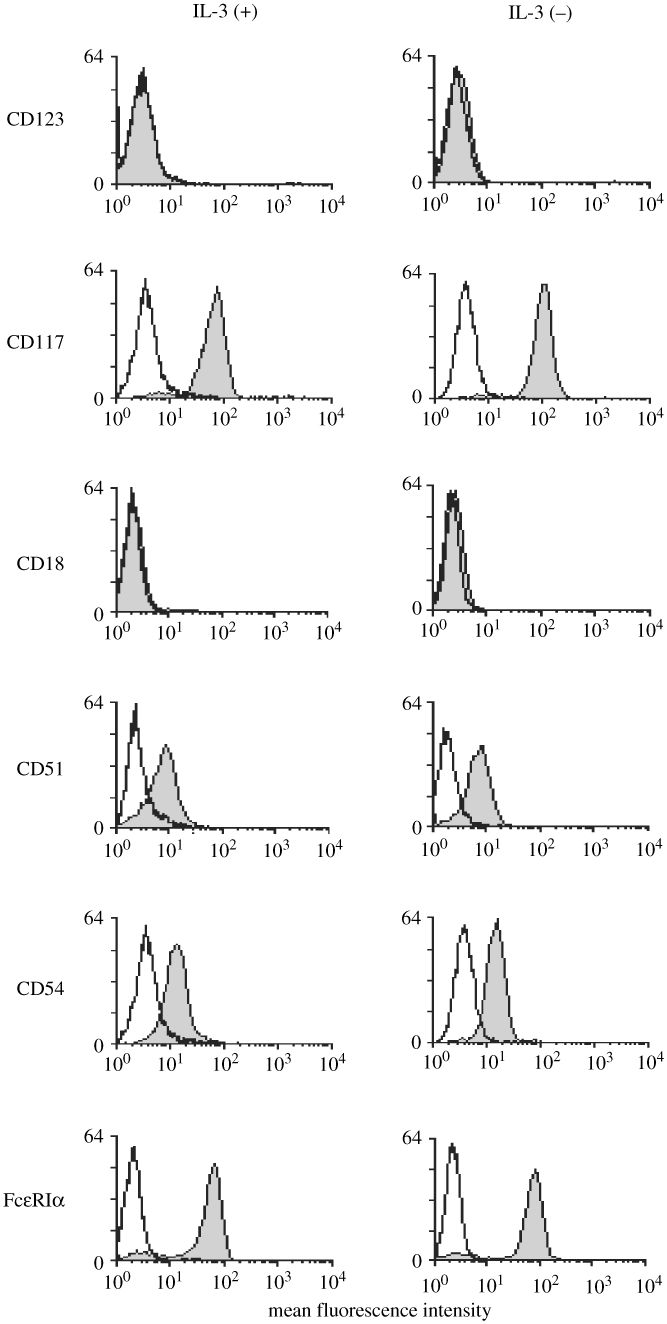
Consistent surface expression of FcεRIα, CD123 and integrins on HBMMC cultured initially either with or without IL-3. HBMMC (either IL-3(+) or IL-3(−)) were labeled with specific mAb against FcεRIα, CD123, CD117 and integrins such as CD18, CD51, and CD54, or subtype-matched irrevalent mouse IgG (shaded area) subsequently labeled with FITC-conjugated rabbit anti-mouse immunoglobulins. Fluorescence intensity was analyzed by flow cytometry. Histograms with specific mAb were overlaid with those using the isotype-matched mouse IgG. Data are representative of three independent experiments that showed similar results.

As reported previously, HBMMC cultured in the presence of rhSCF + rhIL-6 constitutively express FcεRIα on the cell surface, but CD123 (IL-3 receptor) was undetectable (Shimizu, 2002). We therefore compared the expression of these molecules on HBMMC. IL-3 did not alter the expression of FcεRIα and CD123, as shown in Figure 5. The expression of integrins such as CD18, CD51 and CD54 were examined, but there were no significant differences between IL-3 (+) and IL-3 (−) cells.

6. Histamine release from HBMMC by crosslink of FcεRIα in the presence or absence of rhSCF.

**Figure 6:**
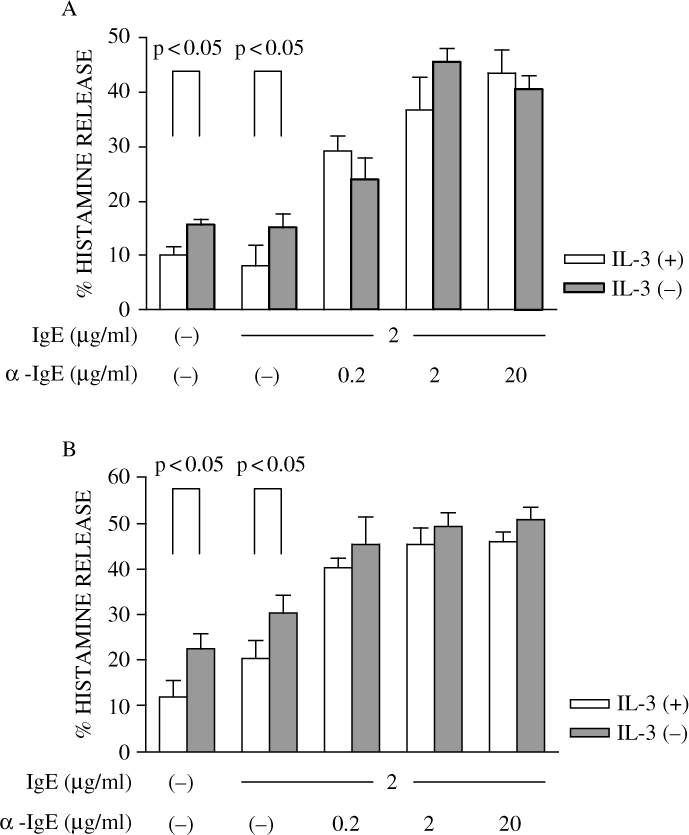
Histamine release by crosslink of FcεRIα. HBMMC (either IL-3 (+) or IL-3 (−)) were sensitized overnight at 37°C with IgE (2 μg/ml) then challenged with anti-IgE (2 μg/ml) for 30 min at 37°C in the absence (A) or presence of 100 ng/ml rhSCF (B). Cells were centrifuged, and then supernatants were collected. Histamine levels in sonicated cells and supernatants were measured and % histamine release was calculated as described in Materials and Methods. Data are the mean ± SEM of 3 independent experiments.

We next evaluated the histamine release from HBMMC. As shown in Figure 6A, spontaneous histamine release (without IgE crosslink) from IL-3 (+) cells was 10.2 ± 1.5%, and 15.6 ± 1.0% from IL-3 (−) cells. When rhSCF was present, 12.1 ± 3.5% of spontaneous histamine release was observed from IL-3 (+), and 22.5 ± 3.4% from IL-3 (−), as shown in Figure 6B. Histamine release by IgE crosslink was dose dependently increased in both IL-3 (+) and IL-3 (−) cells, but there was no significant difference between the groups. Why spontaneous release of histamine from IL-3 (−) cells is higher than that from IL-3 (+) cells remains to be determined.

7. Comprehensive analysis of IL-3-regulated and IgE-challenged gene expression by HBMMC.

To identify genes regulated by IL-3 and aggregation of FcεRI, Gene-Chip analysis was performed. Each signal (average difference) was normalized with the median value of each GeneChip. Approximately 23,000 transcripts were analyzed, and 1,994 transcripts were subjected to clustering analysis after eliminating the transcripts having low levels (less than 3-fold of the median signal) or absent calls. The transcripts were further screened based on the three-fold increase in inactivated or activated with IgE crosslinking between IL-3 (+) and IL-3 (−) cells. Finally, we found 3 and 19 transcripts that were up-regulated or down-regulated in inactivated IL-3 (+) cells, respectively. The 95 and 3 transcripts, respectively, were found to be up-regulated or down-regulated by IgE crosslinking in both IL-3 (+) and IL-3 (−) cells. However, 1 and 2 transcripts, respectively, were up- or down-regulated by IgE crosslink in IL-3 (+) cells as compared to IL-3 (−) cells. 0 and 1 transcript, respectively, were up- or down-regulated by IgE crosslinking in IL-3 (−) cells. There was no mast cell-specific transcript between the cells cultured in the presence or absence of IL-3 and activated/inactivated with IgE crosslinking.

## DISCUSSION

IL-3 is an important growth and differentiation factor for eosinophils and basophils. IL-3 is also an essential growth and differentiation factor for mouse mast cell development *in vitro* (Thompson et al., 1990; Wright, 2006). In contrast, *in vivo*, IL-3 is dispensable for mouse mast cell development, but is required for normal mast cell expansion during immune responses to parasites (Lantz, 1998).

SCF and IL-3 induce signal transduction through several pathways including phospholipase C, Ras-MAP kinase, and Stat5 (Shelburne, 2003) in mouse mast cell development. Of these proteins, Stat5 is important, since it is activated by both SCF and IL-3 (Shelburne, 2002). The absence of Stat5 resulted in a total loss of *in vivo* mast cell development.

In humans, IL-3 is thought to have little effect, if any, on mast cell differentiation (Schwartz, 2002). Recently, IL-3 was used for the culture of human CD34+ cells derived from cord blood, and it is controversial whether IL-3 affects the differentiation of human mast cells. There are two time points for the addition of IL-3 in the culture system. Nakajima et al. added IL-3 to the culture media of mast cells after 11 weeks of culture to prevent apoptosis (Nakajima, 2002). Yanagida et al. found IL-3R on human cord blood-derived cultured mast cells, but it had a negligible effect on IgE/anti-IgE antibody-induced histamine release (Yanagida, 1995).

The other time point for the addition of IL-3 was considered based on the report by Kirshenbaum et al. They added IL-3 to CD34+ cells at the early stage of differentiation, which is commonly recommended for mast cell culture (Saito, 2006). It has also been reported that IL-3 at low concentration can enhance the growth of mast cell colonies without inducing granulocyte or macrophage (GM) colonies in cord blood-derived mast cells (Okayama and Kawakami, 2006). However, whether early stimulation of progenitors with IL-3 affects the differentiation of mast cells has not been well elucidated.

In the experiments shown here, a low concentration of rhIL-3 was added at an early stage to investigate the effect of IL-3 at the precursor level. As shown in Figure 1, total cell number temporarily increased and the appearance of metachromatic cells was reduced when IL-3 was present at the early phase of differentiation, but the final cell number was not significantly different between IL-3 (+) and IL-3 (−) cells. Encabo et al reported that IL-3 was beneficial in the expansion of total cells, CD34+ cells, CD133+ cells, and colony forming units (CFU), but detrimental to the increase of telomerase activity and to the number of long-term culture-initiating cells (LTC-IC) which are the more immature hematopoietic precursors (Encabo, Mateu, Carbonell-Uberos and Minana, 2003). Our data also suggest that the precursor cells increased probably because of the temporal increase in telomerase activity, and that differentiation was reduced in accordance with the decline in telomerase activity.

Our next aim is to see if rhIL-3 affects the structure of human mast cells. As shown in Figure 2, the appearance of granules observed by electron microscopy looked different between IL-3 (+) and IL-3 (−) cells. It is therefore anticipated that the granule content as well as function was different in each cell. Although tryptase mRNA transcript was reduced at 4 weeks of culture in the presence of IL-3, no typical differences in tryptase and chymase protein and mRNA levels were observed at 12 weeks of culture. Surface antigen expression of CD123, CD18, CD51, CD54, and FcεRIα were also not affected by IL-3. CD123 was expressed on 4-week-old cultured human cord blood mast cells in the presence of rhSCF, but 9-week-old cord blood mast cells which showed substantial expression of β3 integrin became CD123-negative. CD123 was expressed on isolated human intestinal mast cells (Gebhardt, 2002), but not on those cells isolated either from lung (Valent, 1990), tonsils (Fureder, 1997), or kidney (Beil, 1998). As shown in Figure 5, HBMMC did not express CD123 when evaluated by FACS. It is therefore hypothesized that mast cell progenitors proliferate in response to IL-3 via CD123, and become less responsive to IL-3 as cells proliferate into CD123 (−) mature mast cells.

Histamine release by crosslink of FcεRIα is not significantly different between the IL-3 (+) and IL-3 (−) groups. However, spontaneous histamine release mediated by exogenous rhSCF was greater in the IL-3 (−) than in the IL-3 (+) group. It is reported that IL-6 enhances spontaneous release of histamine from human peripheral blood-derived cultured mast cells from approximately 2 to 10–15% (Kikuchi, 2002). Although IL-6 was not present in the histamine release assay, membrane-bound IL-6 during culture might affect histamine release. The distinct mechanism for the reduction of histamine release by IL-3 remains to be elucidated.

Finally, GeneChip analysis was performed to see the differences in unknown transcripts. However, no specific genes were detected to show the difference between IL-3 (+) and IL-3 (−) cells in inactivated and activated with IgE crosslink. The addition of a low concentration of IL-3 at the early stage of differentiation was commonly employed to obtain a large number of cultured mast cells. Our concern was whether IL-3 modulates the structure and function of mast cell during culture. These data suggest that mast cell structure and function was not altered by initial activation with IL-3.

In conclusion, IL-3 does not affect the differentiation of human bone marrow mast cells cultured in the presence of SCF and IL-6. IL-3 was recently used and recommended to obtain a large number of cord blood mast cells. Although not increased in human bone marrow system, HBMMC cultured initially with IL-3 are a good tool for studying mast cell biology. Further examination is required for additional findings on the modulation of differentiation and function such as spontaneous histamine release.
